# International Survey of Medical Students Exposure to Relevant Global Surgery (ISOMERS): A Cross-Sectional Study

**DOI:** 10.1007/s00268-022-06440-0

**Published:** 2022-02-01

**Authors:** InciSioN Collaborative, Soham Bandyopadhyay, Ulrick S. Kanmounye, Katayoun S. Madani, Joana Beltrano, Mashkur A. Isa, Reuben Y. K. Ooi, Mamta Swaroop, Sayed S. N. H. Shah, Michal Kawka, Daniel S. Nteranya, Halimah Khalil, Nermin Badwi, Theophilus T. K. Anyomih, Eric Twizeyimana, Hitomi Kimura, Dissan Matovu, Sajibur Rahman, Hamaiyal Sana, Kevouy Reid, Syeda Fatema Alam, Priyansh Shah, Raoul Ndayiragije, Moshi M. Shabani, Victor Ruzibukya, Naeem Abdul Yusuf Patel, Aemon B. Fissha, Poorvaprabha Patil, Nelson Udeme-Abasi, Yuki J. Ng, Aaron Daniel Brake, Abdul Rehman Arshad, Adeola Adekanle, Ahmed Ayman Elmelegy, Aimee Wilkinson, Al Hasnat Turab, Amany Mostafa Taha Kilany, Amar Hadzić, Amina Naeme, Anamaria Pranjić, Andrew Snyder, Anika Shahrin, Anthonia Adefolaju, Anusha Jayaram, Arsen Muhumuza, Arsène Daniel Nyalundja, Asumi Hayashi, Ayaka Oda, Ayuka Kuroki, Bancy Waithera Mbogo, Bathsheba Wariso, Blake M. Hauser, Brea Willey, Chipegwa Mlula, Chloe Jordan, Christabel Phiri, Chunying Selvakumaran, Collins Ighaba Dambo, Connor J. Peck, Cynthia Chukwudi-Oje, Daisy Evans, Daniel David Otobo, Deen Garba, Doreen Kasongi, Edwin M. Mulwanda, Erinfolami Habiba Olarinre, Esther Annang, Fady K. Soliman, Faith Wambui Muchemi, Fatih Ademović, Fidelis Msikwa, Foo Chuan Yi, Gregory Goodluck, Gregory Karelas, Hannah A. Levy, Holly Sprow, Ibrahim Bin Huzaifa, James Lee, Jannatul Ferdous, Kaweesi Henry, Jasmina Suko, Khair Ul Barayya, Khairoon Abdulkadir Mohamed, Kieran Das, Komal Iftikhar, Kota Kurosawa, Kristin Cardiel Nunez, Kyle Langston, Lahin Amlani, Latoya A. Stewart, Leong Kah Chun, Mahmoud Ayman Soliman, Maisha Samiha Binte Akter, Marija Bjeletić, Marta de Andres Crespo, Marwa Saad, Maylander Menard, Md. Fahim Faisal, Mehak Kakwani, Mehr Muhammad Adeel Riaz, Mercellina Nduku Musyoki, Milica Malešević, Miriam Pueschel, Mohamed Adwi, Mohamed Bella Jalloh, Mohamed E. Ghanem, Mohammed Talha Bashir, Momna Sajjad Raja, Monalisa M. J. Faulkner, Moomtahina Fatima, Mubanga Chitalu, Muktasid Al Mubin, Mushila Nguza Armand, Mwaba Kabwe Bizwell, Myoung Hyun Choi, Navid Mahmud Khan, Olaoluwa Adeyemo, Oyindayo Hassan, Pavanraj Singh Chana, Praise Oluwajuwon Stephen, Priyanka Menon, Rieko Miura, Rika Terashima, Robert Zachary Fender, Rokaya Salah Elsayed, Rosie Rayner, Ryo Takahashi, Saad Ilyas, Sakib Hasan, Sallu Dawo, Sameer Saleem Tebha, Samipya Kafle, Sara M. Hussein, Sarah Honjo, Sayed Shah Nur Hussein Shah, Setthasorn Zhi Yang Ooi, Shamsudeen Aliyu, Shahyan Ur Rehman, Shinju Usami, Shion Kachi, Shiraz Shafi, Sulaymaan Al Majid, Syed Ramiz Ahnaf, Syed Zaki Muhammad, Takako Mizuguchi, Tashi Maseland, Wei Xiang Teh, Viraj Shah, Wentin Chen, William Mauya, Won Young Yoon, Yacine Issiou, Xinye Yek, Yoshiki Tsumura, Yurika Nishikawa, Zara Khan

**Affiliations:** https://incisionetwork.org/, Geneva, Switzerland

## Abstract

**Background:**

The principles of global surgery should be taught as a part of the core curriculum in medical schools. The need for medical students to be familiar with the topic is increasing in acceptance. There is, however, a paucity of data on how medical students are exposed to global surgery. This study aims to evaluate exposure of medical students to global surgery, awareness of the key messages of the Lancet Commission on Global Surgery, global surgery career aspirations and barriers to said aspirations.

**Methods:**

ISOMERS was a multi-centre, online, cross-sectional survey of final year medical students globally. The questionnaire utilised a combination of Likert-scale, multiple-choice, and free text questions.

**Results:**

In this study, 1593 final year medical students from 144 medical schools in 20 countries participated. The majority (*n* = 869/1496, 58.1%) believed global surgery to be relevant, despite 17.7% (*n* = 271/1535) having any exposure to global surgery. Most participants (*n* = 1187/1476, 80.4%) wanted additional resources on global surgery. Difficulty in providing appropriate care for patients living abroad (*n* = 854/1242, 68.8%) was the most common perceived barrier to a career in global surgery.

**Conclusions:**

Participants believed global surgery was a relevant topic for medical students and wanted additional resources that they could access on global surgery. It is critical for medical students to become aware that global surgery is a field that aims to address inequity in surgical care not just internationally, but nationally and locally as well.

**Supplementary Information:**

The online version contains supplementary material available at 10.1007/s00268-022-06440-0.

## Introduction

In 2015, the Lancet Commission on Global Surgery released a landmark report highlighting a health disparity faced by over five billion people in the world: a lack of access to safe, timely, and affordable surgical care [1]. Moral and economic arguments for investment into surgery [1–4] led to a relative consensus: surgery is an “indivisible, indispensable part of health care” [1, 5]. A consensus providing the impetus for the formation of dynamic collaborations between high-income countries (HICs) and low-and-middle-income countries (LMICs) [6] with global surgery—“an area of study, research, practice and advocacy that seeks to improve health outcomes and achieve health equity for all people who need surgical and anaesthesia care” [1]—at the heart of the partnerships. In parallel, there have been increasing calls to bolster medical students’ exposure to global surgery from educational organisations [7], trainees [8], and students [9]. Increasing future healthcare practitioner’s understanding, interest, and participation in global surgery is likely to be a critical step towards ensuring global surgical workforce needs are met.

Cross-sectional studies have been conducted across national populations to acquire information on the degree of exposure medical students have to global surgery [9, 10]. These have consistently highlighted medical students have insufficient teaching [9] or experience [10] in global surgery, despite substantial interest in the topic [10, 11]. Global surgery courses and conferences attempt to meet this deficit between supply and demand [11]. However, there is increasing acceptance that the values of global surgery are core principles of medical practice [12, 13]; therefore, some believe global surgery should be mandated to be included in medical school curricula as essential, non-elective modules [14–18]. There is a need to assess whether these beliefs have been translated into policy and whether there is any evidence to advocate for such a change.

There is, however, a paucity of global data on if and how medical students are being exposed to global surgery, globally. In fact, many students may never be exposed to it and could graduate medical school without understanding the meaning of global surgery [19]. The existing data are limited, only available at a national level in three countries [9, 10, 20], decreasing its applicability internationally. Given the pressing need to maintain and increase the global surgery workforce, it is paramount to evaluate if and how medical students are exposed to global surgery. It is equally important to collect data on opinions and knowledge shaped through previous encounters with global surgery [21], and whether these are related to the method of exposure. Therefore, knowledge, career aspirations, and perceived barriers to careers in global surgery need to be clarified at a global level.

The International Survey Of Medical students Exposure to Relevant Global Surgery (ISOMERS) study primarily aimed to evaluate whether medical students were exposed to global surgery during medical school, how they were exposed to global surgery, and whether the types of exposures met the needs of students. Secondary aims of the study included an exploration of students’ awareness of the key messages of the Lancet Global Surgery Commission [1], career aspirations among students, as well as perceived barriers to becoming involved in global surgery. To our knowledge, this is the most comprehensive survey of global surgery exposure to date.

## Methods

### Participants and setting

ISOMERS is a collaborative, international, questionnaire-based cross-sectional survey which was conducted in-line with a pre-specified protocol (see Appendix S1) by InciSioN (International Student Surgical Network). InciSioN is a student and junior-doctor organisation promoting global surgery via research, education and advocacy [22]. The study was delivered by a collaborative of medical students, who acted as regional leads at their medical schools: a model previously used [23].

Medical students were eligible to participate if they were in their final year of a medical school and a regional lead was present at their medical school. Regional leads and individuals involved in the design of this study were excluded.

### Data collection

An initial pilot questionnaire was created based on recommendations from published literature on undergraduate global surgery education [20, 21, 24–27]. This pilot survey was distributed to members of InciSioN who were not involved in study conception or design to gather feedback from the target population of the survey. Based on the feedback, the survey was edited to improve clarity and ensure objectivity. The new survey was subjected to another round of feedback, and further edits were made to develop the final 27-item, self-administered questionnaire (see Appendix S2). The final questionnaire utilised a combination of Likert-scale, multiple choice options, and free text questions to improve the granularity of the data. It collected data on participants’ demographics, exposure to global surgery, awareness of the key messages in the Lancet Global Surgery Commission [1], and career aspirations in global surgery. Previous exposure to global surgery was self-reported based on participants’ perceived past experiences and the definition of global surgery provided.

Regional leads were responsible for identifying final year medical students at their institution and disseminating the questionnaire among them. Regional leads shared the questionnaire with all final year medical students identified at their medical school at least once a week for four consecutive weeks. The questionnaire was administered in English, French, and Japanese. Participants consented to the use of the anonymised results for the purposes of analysis, distribution, and publication. Participants who were unwilling or unable to give consent to the study were excluded. Given the method of distribution, participants’ awareness of InciSioN was elucidated to rule out any systematic bias in recruitment methods.

All aspects of this study were reviewed and approved by the Institutional Review Board of Université Technologique Bel Campus in Kinshasa in The Democratic Republic of Congo.

### Data analysis

Descriptive statistics were reported. Where participants indicated a preference to not answer, responses were removed. Countries were classified by income level using World Bank criteria [28]. Chi-squared analysis was used to assess an association between global surgery exposure of medical students and the World Bank income category of the country of the medical school attended, interest in pursuing global surgery as a career, and familiarity with a global surgery career. Statistical analysis was performed using SPSS 26.0 (IBM, New York, USA).

## Results

**Fig. 1:**
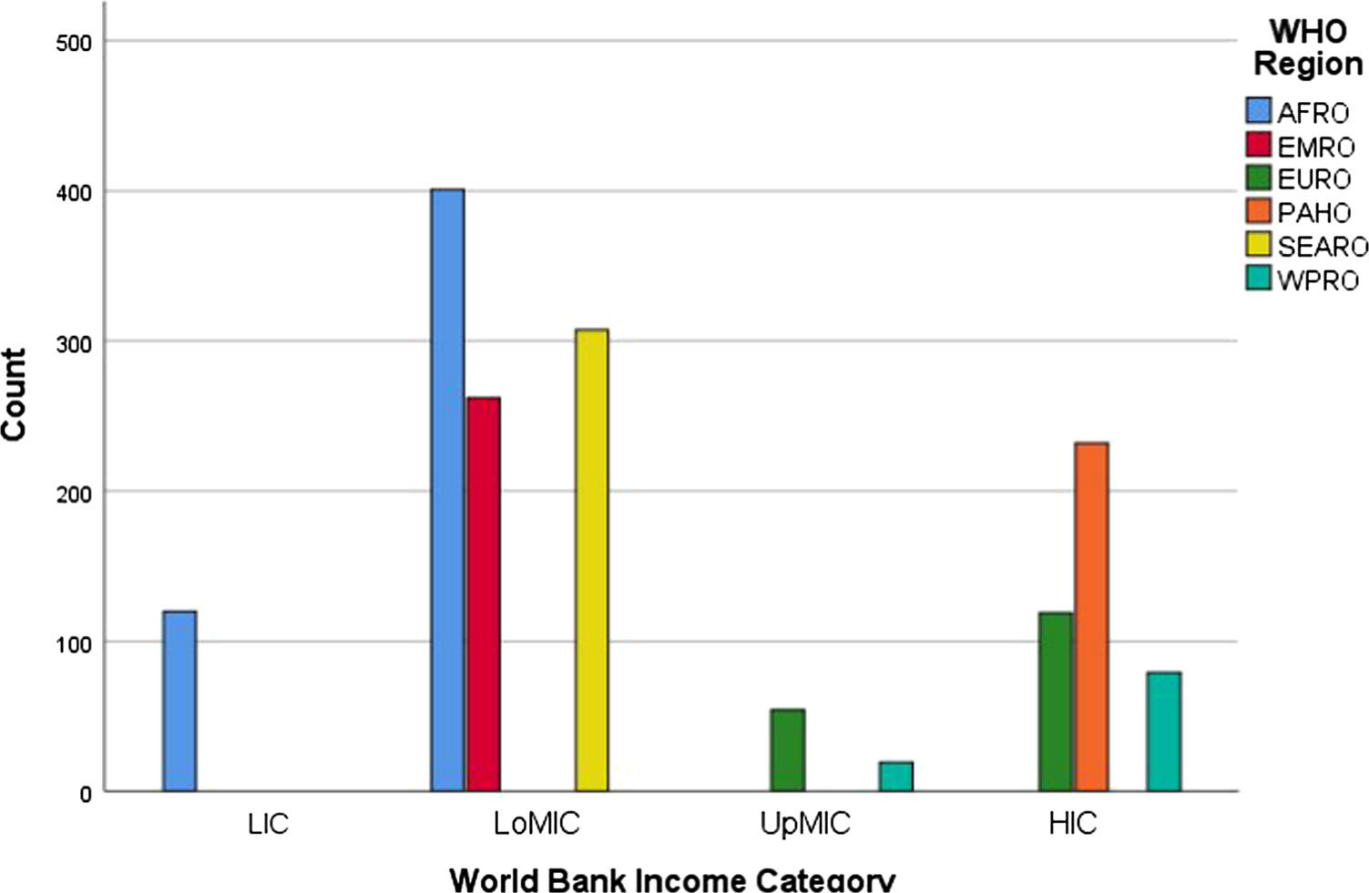
Number of medical students in each World Health Organisation (WHO) region and World Bank Income Category. LIC = low-income country. LoMIC = lower-middle income country. UoMIC = upper-middle-income country. HIC = high-income country. AFRO = African Region. EMRO = Eastern Mediterranean Region. EURO = Europe Region. PAHO = Pan-Americas Region. SEARO = South-East Asia Region. WPRO = Western Pacific Region

A total of 1593 final year medical students from 144 medical schools participated in the ISOMERS study (Table 1). Most of the medical students (*n* = 970/1593, 60.9%) went to a medical school in a lower-middle income country (Fig. 1). A minority of these medical students had heard of InciSioN (*n* = 569/1559, 36.5%), and even fewer had been involved in their activities (*n* = 121/1593, 7.6%).

**Table 1:** Number of final year medical students and the country of their medical school

Country of medical school	Frequency (*n*)
Bangladesh	307
Bosnia and Herzegovina	54
Burundi	9
Democratic Republic of the Congo	23
Egypt	131
Japan	79
Kenya	40
Malaysia	19
Nigeria	132
Pakistan	131
Poland	5
Rwanda	6
Sierra Leone	49
Somalia	2
Somaliland	31
Tanzania	182
Uganda	5
United Kingdom	114
United States of America	232
Zambia	42
Total	1593

**Fig. 2:**
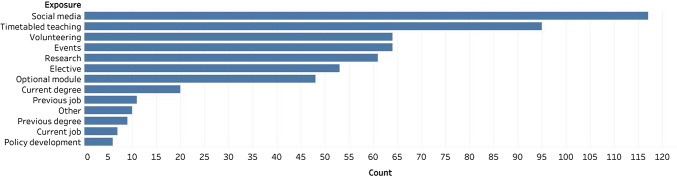
Mechanisms through which individuals gained exposure to global surgery

Most participants (*n* = 869/1496, 58.1%) believed global surgery to be a relevant topic for medical students to know. A minority of respondents (*n* = 271/1535, 17.7%) reported having had exposure to global surgery. Social media (*n* = 117/271, 43%) was the most cited mechanism through which individuals had gained exposure to global surgery (Fig. 2). There was a significant difference in exposure to global surgery based on the income status of a participant’s medical school (*p* = 0.007), with individuals in upper-middle-income countries (*n* = 18/64, 28.1%) being the most likely to be exposed and individuals in lower-middle-income countries (*n* = 143/795, 15.2%) being the least likely to be exposed. Most participants (*n* = 1004/1454, 69.1%) reported the medical school did not offer optional student selected components or elective modules relevant to global surgery.

Among those who had had exposure to global surgery, the majority (*n* = 230/261, 88.1%) wanted more exposure, primarily through events (*n* = 118/230, 51%), online modules (*n* = 104/230, 45%), and timetabled teaching (*n* = 101/230, 44%). These findings were reflected among all participants; a majority (*n* = 1187/1476, 80.4%) wanted additional resources on global surgery, but there were a minority (*n* = 609/1470, 41.4%) who thought there should not be more compulsory timetabled teaching related to global surgery during medical school.

Only 166 participants (*n* = 166/1438, 11.5%) reported that global surgery had been assessed at their medical school, with a minority of participants correctly answering knowledge based questions on global surgery (Table 2).

**Table 2:** Percentage of participants who correctly answered various questions related to global surgery

Question	Percent who answered correctly (*n* = /)
Which of these is not a bellwether surgical procedure?	50.4% (*n* = 624/1238)
What is considered to be timely access to a bellwether surgical procedure?	28.3% (*n* = 345/1218)
Approximately how many people around the world lack access to timely safe, affordable surgical and anaesthesia care when needed?	19.5% (*n* = 235/1206)
Approximately how many additional surgical procedures each year could keep mortality and morbidity to a minimum worldwide?	29.4% (*n* = 349/1186)
Which of the following do not form part of the definition for catastrophic out-of-pocket payments?	64.9% (*n* = 757/1167)
Approximately how many individuals worldwide face catastrophic health expenditure due to payment for surgery and anaesthesia each year?	10.8% (*n* = 126/1164)
How many disability-adjusted life-years could be averted each year through provision of basic surgical services?	27.9% (*n* = 323/1156)

**Fig. 3:**
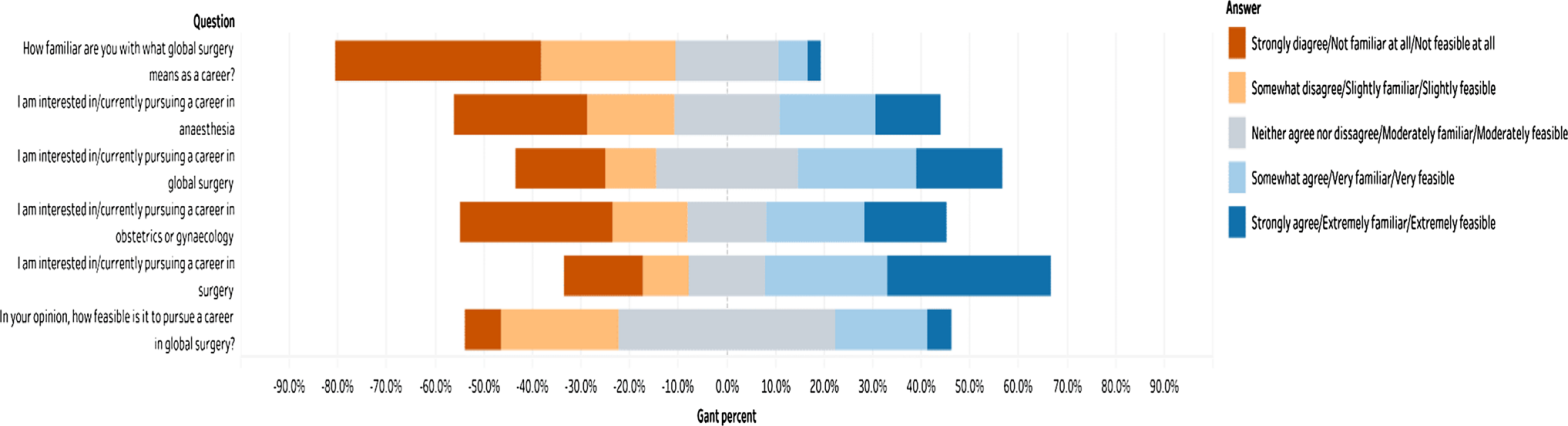
Likert-scale responses to questions about interest, familiarity, and perceived viability of a global surgery career

Most of the respondents (*n* = 743/1264, 58.8%) were interested in or were actively pursuing a career in surgery. Fewer respondents (*n* = 483/1149, 42.1%) stated they were interested in or were actively pursuing a career in global surgery. This was significantly associated with whether an individual had exposure to global surgery (*p* < 0.001) with individuals that reported having had exposure to global surgery more likely to be interested in or actively pursuing a career in global surgery. A minority of participants reported being moderately familiar (*n* = 273/ 1287, 21.2%), very familiar (*n* = 78/ 1287, 6.1%), or extremely familiar (*n* = 34/ 1287, 2.6%) with the requirements for a global surgery career (Fig. 3). Individuals who had exposure to global surgery were significantly more likely to report being familiar with a global surgery career (*p* < 0.001). A minority of respondents believed a global surgery career was not feasible at all (*n* = 91/1245, 7.3%). This belief was significantly associated with not having exposure to global surgery (*p* < 0.001). A majority of participants (*n* = 1242/1593, 78%) cited a number of potential barriers to pursuing a career in global surgery (Table 3).

**Table 3:** Perceived barriers to pursuing a career in global surgery

Barriers to pursuing a career in global surgery	Percentage of participants (*n* = /1242)
Increased length of training	55.3% (687)
Lack of established career paths in global surgery	43.0% (534)
Lack of surgical role models and mentorship in global health	52.8% (656)
Constrained time to travel abroad during one’s career	55.8% (693)
Difficulty with providing appropriate care for patient living abroad	68.8% (854)
Ethical issues with providing surgical care in resource-limited communities	62.6% (777)
Financial constraints	38.8% (482)

## Discussion

### Key findings

Participants believed global surgery was a relevant topic for medical students to know and wanted additional resources to learn about global surgery. Participants reported a reliance on non-medical school sources, such as social media, to gain exposure to the topic. There was a general consensus to increase teaching in medical schools, either compulsory or optional, among the participants. If the recommendation of the students was to be incorporated into medical student curricula, this would improve the gap in knowledge regarding surgical inequity worldwide and enlighten the future of the operating room to the feasibility of surgical care in low resource setting in HICs and LMICs.

### Implications

Given the large number of participants citing travelling abroad or working abroad as a barrier to pursuing a career in global surgery, it is critical that medical students become more familiar with the definition of global surgery: *an area of study, research, practice and advocacy seeking to improve health outcomes and achieve health equity for all people who need surgical and anaesthesia care* [1, 5]. Global surgery is not a field confined to countries other than one’s home nation. It is a field that considers all people; this includes all individuals in HICs and LMICs. Given that this definition preceded the questions in the study, it may be this definition came across as too nebulous to understand. In this case, it is pertinent to introspect as to how better to explain what global surgery is. There have been recent attempts to dissect the original definition to provide necessary granularity [29], but work still needs to be done to advocate the paramount point: global surgery is a field aiming to address inequity in surgical care, not just internationally, but nationally and locally as well. The lack of understanding may also explain why more than half of participants wanted to pursue a career in surgery, but less than half of participants want to pursue a career in global surgery. It is highly unlikely that several final year medical students wishing to pursue surgery are uninterested in optimising surgical care for all their future patients; the issue instead is likely to be one of not being familiar with what global surgery means as a career. This is not just an issue that pervades medical students; healthcare professionals are also prone to this misconception [19, 30]. In HICs, global surgery was often interpreted as surgical work in LMICs [19, 31]. This misconception has resulted in previous reviews on global surgery led by junior researchers in HICs erroneously narrowing their focus to work conducted in LMICs, which has necessitated updates to be made to the existing body of literature [32]. In LMICs, global surgery is thought to be a new term that encapsulates previous work being done under the moniker of rural surgery in LMICs and brings international support for these endeavours [30, 33]. This again comes with its own issues of resources being diverted away from under-served populations in HICs [29]. Early exposure to global surgery in medical school may also ameliorate these misconceptions among health professionals, as our study suggests individuals who had exposure to global surgery were significantly more likely to report being familiar with a global surgery career.

Scepticism of global surgery is not a new phenomenon [34]. The solution for this has long been recognised to be more education on global surgery in medical schools [9, 34]. Fortuitously, most participants were keen for this to happen too, and there have been similar findings in previous national studies [10, 20]. However, given that global surgery was reported by most of our participants not to be assessed at their medical school, simply teaching about global surgery is unlikely to fix this issue. Teaching without assessment will generally be viewed as non-compulsory, particularly by busy medical students [35]. Therefore, efforts in this disciple should be directed towards the development and integration of global surgery modules into courses provided within the medical school curricula, which aim to provide a core understanding of the subject matter along with evaluation of higher levels of learning in implementation. A template for a global surgery curriculum has been published in the Lancet based on work conducted by the Global Health Learning Outcomes Working Group [14].

Our study also hints at the need for greater mentorship for aspiring global surgery candidates. This can be tackled by organisations working in global surgery, such as Global Initiative for Children’s Surgery, Association for Academic Surgery Global Affairs Committee, and InciSioN to name a few. Given the number of surgeons with a passion for mentoring associated with InciSioN [22] and similar global health organisations, the onus is on us to reach out to students—who may or may not know what global surgery really is—and provide them with the necessary education and career guidance. The feasibility of global surgery as a career may be greatly enhanced by its transparent promotion to medical students by like-minded individuals who are passionate about health equity [36,37].

### Limitations

Our key finding of the lack of knowledge about what global surgery is among our participants also introduces a significant limitation into how our study results should be interpreted. If individuals are unable to recognise global surgery, they may be unable to recognise teaching or assessment around global surgery. Similarly, their desire for more global surgery exposure may be based around their desire for greater knowledge of surgical systems internationally, rather than all aspects of global surgery. However, at the same time this finding also reassures us that this study being conducted by InciSioN did not introduce a systematic bias of capturing purely the views of students interested in pursuing a career in global surgery. Although given the study's name, which included the phrase “global surgery”, there may have been a selection bias, resulting in students interested in global health, global surgery, or surgical careers being more likely to respond. However, this does not negate the findings, which highlighted the need for more global surgery teaching as well as the importance of exposure to global surgery. It should be noted, however, that there was an inequitable distribution of participants from HICs and LMICs. Approximately two-thirds of all participants were based in LMICs. However, given the lack of presence of individuals from LMICs in most studies on global surgery, these data points provide a novel opportunity to assess global surgery-related knowledge, career aspirations, and views on the quality of global surgery education being offered.

## Conclusions

The collaborative model of InciSioN and the strong presence of LMICs students in the study, demonstrates a clear interest from medical students in learning about and in pursuing global surgery education and research. The current approach to global surgery education does not sufficiently equip medical students with the knowledge and skills needed. The ISOMERS study gauged the knowledge and attitudes of medical students towards global surgery and highlighted the need for the development and integration of a global surgery education module or course to medical school curricula.

## Supplementary Information

Below is the link to the electronic supplementary material.

Supplementary file1 (DOCX 19 kb)

Supplementary file2 (PDF 1924 kb)

## Data Availability

The datasets used and analysed during the current study are available from the corresponding author on reasonable request.

## References

[1] Meara JG, Leather AJM, Hagander L (2015). Global Surgery 2030: evidence and solutions for achieving health, welfare, and economic development. Lancet.

[2] Price R, Makasa E, Hollands M (2015). World health assembly resolution WHA68.15: “strengthening emergency and essential surgical care and anesthesia as a component of universal health coverage” - addressing the public health gaps arising from lack of safe, affordable and accessible surgical a. World J Surg.

[3] World Health Organization (1980) Address by Dr H. Mahler Director-General of the World Health Organization. https://www.who.int/surgery/strategies/Mahler1980speech.pdf. Accessed 1 Apr 2020

[4] Jamison DT, Gelband H, Horton S (2017). Disease Control Priorities: Improving Health and Reducing Poverty.

[5] Dare AJ, Grimes CE, Gillies R (2014). Global surgery: defining an emerging global health field. Lancet.

[6] The Royal Colleges of Surgeons in the U.K. and Ireland The Royal Colleges of Surgeons in the U.K. and Ireland: A common vision for global surgery | The Bulletin. https://bulletin.facs.org/2018/05/the-royal-colleges-of-surgeons-in-the-u-k-and-ireland-a-common-vision-for-global-surgery/. Accessed 18 Jun 2020

[7] The Royal College of Surgeons England, (RCSENG). (2015) National undergraduate curriculum in surgery 2015. RCSENG – Prof Stand Regul

[8] Patel R, Khundkar R, Peter N (2019). Improving global surgery education for trainees. Int J Surg Glob Heal.

[9] Collaborative IU (2020). Global health education in medical schools (GHEMS): a national, collaborative study of medical curricula. BMC Med Educ.

[10] Kanmounye US, Mbonda AN, Djiofack D (2020). Exploring the knowledge and attitudes of Cameroonian medical students towards global surgery: a web-based survey. PLoS ONE.

[11] Gosselin-Tardif A, Butler-Laporte G, Vassiliou M (2014). Enhancing medical students’ education and careers in global surgery. Can J Surg.

[12] Medical Schools Council (2014) The consensus statement on the role of the doctor. http://www.medschools.ac.uk/Publications/Pages/Role-of-the-doctor-consensus-statement-2014.aspx. Accessed 17 Feb 2020

[13] General Medical Council (1995) Duties of a doctor (confidentiality). https://www.gmc-uk.org/ethical-guidance/ethical-guidance-for-doctors/good-medical-practice/duties-of-a-doctor. Accessed 17 Feb 2020

[14] Johnson O, Lou BS, Willott C (2012). Global health learning outcomes for medical students in the UK. Lancet.

[15] General Medical Council (2009) Tomorrow’s Doctors - Outcomes and standards for undergraduate medical education. In: 2009

[16] General Medical Council Generic professional capabilities framework guidance on implementation for colleges and faculties. https://www.gmc-uk.org/-/media/documents/generic-professional-capabilities-implementation-guidance-0517_pdf-70432028.pdf/. Accessed 17 Feb 2020

[17] General Medical Council Outcomes for graduates 2018. https://www.gmc-uk.org/-/media/documents/dc11326-outcomes-for-graduates-2018_pdf-75040796.pdf. Accessed 17 Feb 2020

[18] Brewer TF, Saba N, Clair V (2009). From boutique to basic: A call for standardised medical education in global health. Med Educ.

[19] Abraham MN, Abraham PJ, Chen H, Hendershot KM (2020). What is global surgery? Identifying misconceptions among health professionals. Am J Surg.

[20] Scott EM, Fallah PN, Blitzer DN (2019). Next generation of global surgeons: aligning interest with early access to global surgery education. J Surg Res.

[21] Mehta A, Xu T, Murray M, Casey KM (2017). Medical student perceptions of global surgery at an academic institution: identifying gaps in global health education. Acad Med.

[22] (2020) InciSioN – International Student Surgical Network. https://incisionetwork.org/. Accessed 28 Jan 2021

[23] Bandyopadhyay S, Shortland T, Wadanamby SW (2019). Global health education in UK medical schools (GHEMS) study protocol. J Glob Heal Reports.

[24] Ehn S, Agardh A, Holmer H (2015). Global health education in Swedish medical schools. Scand J Public Health.

[25] Barton A, Williams D, Beverldge M (2008). A survey of Canadian general surgery residents’ interest in international surgery. Can J Surg.

[26] Marks IH, Diaz A, Keem M (2020). Barriers to women entering surgical careers: a global study into medical student perceptions. World J Surg.

[27] Smith F, Lambert TW, Goldacre MJ (2015). Factors influencing junior doctors’ choices of future specialty: trends over time and demographics based on results from UK national surveys. J R Soc Med.

[28] World Bank (2021) World Bank Country and Lending Groups. https://datahelpdesk.worldbank.org/knowledgebase/articles/906519-world-bank-country-and-lending-groups. Accessed 10 Jul 2021

[29] Bath M, Bashford T, Fitzgerald JE (2019). What is 'global surgery’? Defining the multidisciplinary interface between surgery, anaesthesia and public health. BMJ Glob Heal.

[30] Mohan M, Gadgil A, Roy N (2020). Unpacking ‘Global Surgery’: voices from the grassroots. Am J Surg.

[31] Veerappan VR, Jindal RM (2021). Community participation in global surgery. BMJ Glob Heal.

[32] Ravi K, Bentounsi Z, Tariq A (2021). Systematic analysis of authorship demographics in global surgery. BMJ Glob Heal.

[33] Raykar N, Mukhopadhyay S, Saluja S (2019). Implementation of the lancet commission on global surgery in India. Healthcare.

[34] Fallah PN, Bernstein M (2019). Barriers to participation in global surgery academic collaborations, and possible solutions: a qualitative study. J Neurosurg.

[35] Havnes A (2002) Examination and learning: an activity-theoretical analysis of the relationship between assessment and learning

[36] Larrazabal AJ, Nieto N, Peterson V (2020). Gender imbalance in medical imaging datasets produces biased classifiers for computer-aided diagnosis. Proc Natl Acad Sci U S A.

[37] Bandyopadhyay S, Moudgil-Joshi J, Norton EJ (2020). Motivations, barriers, and social media: a qualitative study of uptake of women into neurosurgery. Br J Neurosurg.

