# Supplementary material for: International Survey of Medical Students Exposure to Relevant Global Surgery (ISOMERS): A Cross-Sectional Study
Source: World J Surg. 2022 Feb 1;46(7):1577–84. doi: 10.1007/s00268-022-06440-0 (PMC9174132; doi:10.1007/s00268-022-06440-0)
Supplement: Supplementary file 1 — Supplementary file1 (DOCX 19 kb) [file 268_2022_6440_MOESM1_ESM.docx]

**Appendices**

*Appendix S1*

**Summary**

To our knowledge, there is scarce data evaluating the extent of global surgery opportunities at medical schools across the world. Given the International Student Surgical Network (InciSioN) has national working groups (NWGS) across the world, we are in a unique position to establish the global surgery educational practices globally. Establishing an international framework on global surgery exposure and knowledge is important, as it will enable InciSioN to provide support and supplement formal educational practices where needed. This online, international, multi-centre, questionnaire-based study evaluates global surgery exposure, global surgery knowledge and career aspirations among medical students across the world.

**Methods**

Primary aim:

To collect high quality and relevant data regarding global surgery exposure for medical students across the world in order to establish an international framework

Secondary Aims:

To examine students’ awareness of the key messages of the Lancet Global Surgery Commission

To characterise the proportion of medical students who would want to be involved in global surgery in their future career

To ascertain perceived barriers to becoming involved in global surgery

Study Design

The International Survey Of Medical students Exposure to Relevant global Surgery (ISOMERS) study is an online, international, multi-centre, questionnaire-based study evaluating global surgery exposure and career aspirations among final year medical students across the world. Data will be collected on the demographics of participants, their exposure to global surgery, their awareness of the key messages of the Lancet Global Surgery Commission, and their career aspirations. The questionnaire will be disseminated by email and social media through collaborative university medical school and student networks, such as the network of InciSioN National Working Groups (NWGs) across the world. The generic collaborative method, and the benefits that participating students derive from it are based on previous well-documented literature.

Study Population

All medical schools across the world will be eligible to participate. Participant eligibility will be determined according to pre-specified criteria. Responses will be excluded if the participant did not meet the inclusion criteria or met the exclusion criteria. Participation rates will be calculated as the total number of eligible participants over the total number of final year medical students at participating medical schools. The minimum sample size required from our target population for a margin of error of 5% and a confidence level of 95% is 377 assuming a 50% response distribution for each question.

Inclusion criteria:

All final year medical students across the world

Exclusion criteria:

Listed as a collaborative author or named author on any publications from this study.

Primary Outcomes

The proportion of students that have had exposure to global surgery in each country.

The range of modalities utilised by medical students to gain exposure to global surgery.

The percentage of students that prefer each modality.

Secondary Outcomes

The proportion of students aware of the key messages of the Lancet Global Surgery Commission

The proportion of students who do not want to pursue a career in global surgery.

The range of barriers identified by medical students to pursuing a career in global surgery.

Methods for recruiting participants

All final year medical students at any medical school in the world will be eligible to participate. Medical students will be invited to participate in the study by email and social media.

In addition, final year medical students enrolled in any medical school in the world may be invited to collaborate in the study as regional leads as described in Table 1. Collaborators will ensure that the steering committee possess accurate data about the number of final year medical students at their institution, and they will be primarily responsible for disseminating this questionnaire amongst students at their medical school.

Financial and other rewards to participants

No financial or other rewards will be offered to participants.

Data collection

ISOMERS will be delivered by NWGs of InciSioN. Each NWG will be responsible for conducting ISOMERS within their country and disseminating the questionnaire. Medical students enrolled in any medical school may be invited to collaborate in this study as regional leads who are coordinated by the appropriate NWG. The role of a regional lead would be to identify the number of final year medical students at their institution and to disseminate the questionnaire among students at their medical school. Dissemination will be done using any online tools such as social media and mailing lists. The questionnaire must be disseminated by regional leads using all online tools available to them at least once a week for 6 consecutive weeks. Regional leads must share evidence to show that they have done to this to their appropriate NWG to be awarded collaborator status within our collaborative authorship model.

The online questionnaire utilises a combination of the Likert scale, multiple choice options and free text questions in order to broaden the capture and improve granularity of the data. The questionnaire is divided into four major sections: background information, provision of global surgery teaching, knowledge about global surgery and career aspirations. All sections of the questionnaire must be completed for the answers to be deemed eligible. Following data collection, the data will be validated and analysed by the steering committee. The questionnaire has been sent to multiple medical students who were not involved in creating the data collection proforma. Their responses were evaluated for potential problems, and the questionnaire was updated to ensure the questions were relevant, comprehensive and accessible.

Medical schools will be able to request for their own specific data and the analysis done on said data from the ISOMERS steering committee following study completion. InciSioN NWGs will be able to request for their country specific de-identified and pseudo-anonymised data.

Information Governance

No identifiable information will be collected. No individual medical school data will be published with the exception of case studies with permission from the medical school. Data will be collected using a structured standard questionnaire. All data will be pseudo-anonymised. Providing personally identifying data (i.e. contact details) will be optional and will not be linked to the questionnaire answers given. All other data collected may be used for research purposes. Data will be stored securely and accessible only by the primary researchers and users nominated by them. At the end of the project, the data will be stored for five years after final publication.

Statistical Analysis

The resultant data will be analysed via descriptive statistics. For quantitative data where possible, ANOVA tests will be used for parametric data and the Wilcoxon signed-rank test will be used for non-parametric data. For Likert scale data, a multinomial regression will be utilised.

Authorship

In accordance with National Research Collaborative (NRC) authorship guidelines[39] all publication outputs from ISOMERS will be listed under a unified corporate authorship: ‘International Student Surgical Network**’**. Certain publications will include named authors on the bye-line as well as the group name. Anyone who has demonstrated satisfactory completion of the minimum requirements for authorship will be eligible for PubMed-citable collaborative authorship in accordance with the roles defined below:

**Steering Committee:** Responsible for the protocol design, project coordination, and data handling.

**Writing Group:** Responsible for the overall scientific content, data analysis, and preparation of research manuscripts.

**Collaborators:** A network of medical students across all medical schools. They are responsible for leading the study regionally.

**Ethics and dissemination**

Ethics

Ethical exemption received from the Universite Technologique, “Bel Campus”, University of Technology

Dissemination

The protocol will be disseminated primarily through recruited medical student collaborators. Should medical schools wish to see the protocol, collaborators may pass it along as well.

The survey findings will be presented at local, regional, national, and international conferences by medical student collaborators. A standard PowerPoint presentation and poster will be created for this purpose. All presentations will be coordinated by the ISOMERS steering committee to avoid duplications and to ensure all conference regulations are fulfilled. In addition, the results will be disseminated via publication in a peer-reviewed medical journal. All collaborators will be co-authors of resulting presentations and PubMed Citable co-authors of resulting publication(s).

Following publication, the manuscript can be shared by collaborators with their medical schools to feedback the study results. Medical schools can request for their own specific data and the analysis done on said data from the ISOMERS steering committee following study completion. The fully anonymised dataset will be made publicly available.

**Project Timeline**

|  | **September** | **October** | **November** | **December** | **January** |
| --- | --- | --- | --- | --- | --- |
| Regional Lead Recruitment |  |  |  |  |  |
| Data collection |  |  |  |  |  |
| Data analysis |  |  |  |  |  |
| Manuscript preparation |  |  |  |  |  |

**Table 1:**Project timeline. Extended data collection periods may be incorporated to grant flexibility to centres that may have experienced logistical obstacles to study commencement.

**Conclusion**

The International Survey Of Medical students Exposure to Relevant global Surgery (ISOMERS) will establish whether medical students are exposed to global surgery during medical school, how they are exposed to global surgery, whether the types of exposures meet the needs of students, current global surgery knowledge among students, and career aspirations among students and doctors. This is the first such an endeavour has been undertaken internationally. Our results will allow us to draw comparisons between countries.

*Appendix S2*

PDF attachment
