# Supplementary material for: International Survey of Medical Students Exposure to Relevant Global Surgery (ISOMERS): A Cross-Sectional Study
Source: World J Surg. 2022 Feb 1;46(7):1577–84. doi: 10.1007/s00268-022-06440-0 (PMC9174132; doi:10.1007/s00268-022-06440-0)
Supplement: Supplementary file 2 — Supplementary file2 (PDF 1924 kb) [file 268_2022_6440_MOESM2_ESM.pdf]

English

Default Question Block

International Survey Of Medical students Exposure to Relevant global Surgery (ISOMERS): a cross-sectional study

General Information

The aim of this study is to collect high quality and relevant data regarding global surgery exposure for medical students across the world in order to establish an international framework

We appreciate your interest in participating in this questionnaire. You have been invited to participate as you are 18 years of age or over, and a final year medical student. Please read through this information before agreeing to participate by ticking the ‘yes’ box below.

You may ask any questions before deciding to take part by contacting the national working group (contact details below) of the body carrying out this research study: The International Student Surgical Network (InciSioN).

You will be asked to answer questions about global surgery and your career aspirations. This should take about 10 minutes. No background knowledge is required. The data collected will be used for research purposes. Data will be stored securely and accessible only by the primary researchers and users nominated by them. At the end of the project, the data will be stored for five years after final publication.

Do I have to take part?

Please note that your participation is voluntary. If you do decide to take part, you may withdraw at any point during the questionnaire for any reason before submitting your answers by pressing the closing the browser.

How will my data be used?

Your answers will be completely anonymous, and we will take all reasonable measures to ensure that they remain confidential.

Your data will be stored in a password-protected file and may be used in academic publications. Your IP address will not be stored. All questions are optional. Research data will be stored for a minimum of three years after publication or public release. The data that we collect from you may be transferred to, stored and/or processed at a destination outside your country and your continent. By submitting your personal data, you agree to this transfer, storing or processing.

Who will have access to my data?

Qualtrics is the data controller with respect to your personal data and, as such, will determine how your personal data is used. Please see their privacy notice here: https://www.qualtrics.com/privacy-statement. Qualtrics will share only fully anonymised data with the University of Oxford, for the purposes of research.

We would also like your permission to use your anonymised data in future studies, and to share data with other researchers (e.g. in online databases). Any personal information that could identify you will be removed or changed before files are shared with other researchers or results are made public.

This project has been reviewed by and received ethics exemption through the Université Technologique Bel Campus

Who do I contact if I have a concern about the study or I wish to complain?

If you have a concern about any aspect of this study, please speak to Soham Bandyopadhyay at soham.bandyopadhyay@st-hildas.ox.ac.uk and we will do our best to answer your query. We will acknowledge your concern within 10 working days and give you an indication of how it will be dealt with.

Who to contact for further details?
For any further questions or more information on the study, please contact us on the following email address: soham.bandyopadhyay@st-hildas.ox.ac.uk.

Please note that you may only participate in this survey if you are 18 years of age or over.

○ I certify that I am 18 years of age or over

If you have read the information above and agree to participate with the understanding that the data (including any personal data) you submit will be processed accordingly, please check the relevant box below to get started.

○

Yes, I agree to take part

Block 1

1. BACKGROUND INFORMATION

Are you a final year medical student?

- ○ Yes
- ○ No

Which country is your medical school located in?

[▾]

What is the name of your medical school?

[▾]

What is the title of your degree?

[▾]

How old are you?

What is your gender?

- Male
- Female
- Non-binary
- Prefer to self-describe (please specify)
- Prefer not to answer

Block 2

2. Global Surgery Perceptions

Global Surgery is an "area of study, research, practice, and advocacy that seeks to improve health outcomes and achieve health equity for all people who need surgical and anaesthesia care.".
Meara JG, Greenberg SL.The Lancet Commission on Global Surgery global surgery 2030: evidence and solutions for achieving health, welfare and economic development. Surgery 2015; 157:834–35.

Have you heard of the International Student Surgical Network (InciSioN)?

- Yes
- No

Have you been involved in their activities?

- ○ Yes
- ○ No

Have you had exposure to Global Surgery?

- ○ Yes
- ○ No

Have you gained exposure through any of the following methods (please select all that apply)?

- ☐ Timetabled teaching at medical school
- ☐ Optional module/programme selected at medical school
- ☐ Previous degree
- ☐ Current degree (if not studying for a degree in medicine)
- ☐ Elective
- ☐ Events
- ☐ Social Media
- ☐ Research
- ☐ Policy development
- ☐ Volunteering
- ☐ Previous job
- ☐ Current job
- ☐ Other

Please define what you meant by other?

Are you interested in gaining more exposure to Global Surgery?

- Yes
- No

What would be your preferred methods of gaining more exposure to Global Surgery (please select all that apply)?

- Timetabled teaching at medical school
- Optional module/programme selected at medical school
- Previous degree
- Current degree (if not studying for a degree in medicine)
- Elective
- Online course/modules
- Events (i.e. conferences, workshops, webinars etc.)
- Social Media
- Research
- Policy development
- Volunteering
- Previous job

- ☐ Current job
- ☐ Other

Please define what you meant by other?

____

Are you interested in gaining exposure to Global Surgery?

- ○ Yes
- ○ No

What would be your preferred methods of gaining exposure to Global Surgery (please select all that apply)?

- ☐ Timetabled teaching at medical school
- ☐ Optional module/programme selected at medical school
- ☐ Previous degree
- ☐ Current degree (if not studying for a degree in medicine)
- ☐ Elective
- ☐ Online course/modules
- ☐ Events (i.e. conferences, workshops, webinars etc.)
- ☐ Social Media
- ☐ Research
- ☐ Policy development

- ☐ Volunteering
- ☐ Previous job
- ☐ Current job
- ☐ Other

Please define what you meant by other?

Is Global Surgery a relevant topic for medical students to know about?

- ○ Yes
- ○ Maybe
- ○ No

Should there be more compulsory timetabled teaching relating to global surgery during medical school?

- ○ Yes
- ○ Maybe
- ○ No

Would you like additional resources on global surgery?

- ○ Yes
- ○ No

What form should these resources take (please select all that apply)?

- ☐ Lectures
- ☐ Workshops
- ☐ E-learning
- ☐ Webinar
- ☐ Career Guidance
- ☐ Elective Opportunities
- ☐ Degree Opportunities
- ☐ Other

Please define what you meant by other?

Were there optional student selected components or elective modules, relevant to global surgery, offered as part of your medical school course?

- ○ Yes
- ○ No

Was global surgery assessed at your medical school?

- ○ Yes

○ No

How was it assessed (please select all that apply)?

☐ Essay examination

☐ Essay coursework

☐ Oral examination

☐ OSCE

☐ Short answer questions

☐ Single best answer questions

☐ Other

Please define what you meant by other?

Do medical schools need to have more compulsory timetabled teaching on global surgery?

○ Yes

○ Maybe

○ No

Block 3

3. Knowledge about Global Surgery

Please answer the following to the best of your knowledge:

Which of these is **not** a bellwether surgical procedure?

- ○ Caesarean section
- ○ Laparotomy
- ○ Open Fracture treatment
- ○ Decompression of traumatic brain injury

What is considered to be timely access to a bellwether surgical procedure?

- ○ Within 1 hour
- ○ Within 2 hours
- ○ Within 3 hours
- ○ Within 4 hours
- ○ Within 5 hours
- ○ Within 6 hours

Approximately how many people around the world lack access to timely safe, affordable surgical and anaesthesia care when needed?

- ○ 50 thousand
- ○ 500 thousand
- ○ 5 million

○ 50 million

○ 500 million

○ 5 billion

Approximately how many additional surgical procedures each year could keep mortality and morbidity to a minimum worldwide?

○ 1.43 million

○ 14.3 million

○ 143 million

○ 1.43 billion

○ 14.3 billion

○ 143 billion

Which of the following do **not** form part of the definition for catastrophic out-of-pocket payments?

○ Payments that exceed 10% of total income

○ Payments that are a private expenditure of an individual

○ Payments that exceed 40% of income after basic needs for food and shelter are met

○ Payments that result in an individual going below the relative poverty line

Approximately how many individuals worldwide face catastrophic health expenditure due to payment for surgery and anaesthesia each year?

○ 33 thousand

○ 330 thousand

- 3.3 million
- 33 million
- 330 million
- 3.3 billion

How many disability-adjusted life-years could be averted each year through provision of basic surgical services?

- 772 thousand
- 7.72 million
- 77.2 million
- 772 million
- 7.72 billion
- 77.2 billion

Block 4

4. Career Choices

To what extent do you agree with the following statements

	Strongly agree	Somewhat agree	Neither agree nor disagree	Somewhat disagree	Strongly disagree
I am interested in/currently pursuing a career in surgery	○	○	○	○	○

	Strongly agree	Somewhat agree	Neither agree nor disagree	Somewhat disagree	Strongly disagree
I am interested in/currently pursuing a career in anaesthesia	○	○	○	○	○
I am interested in/currently pursuing a career in obstetrics or gynaecology	○	○	○	○	○
I am interested in/currently pursuing a career in global surgery	○	○	○	○	○

How familiar are you with what global surgery means as a career?

- ○ Extremely familiar
- ○ Very familiar
- ○ Moderately familiar
- ○ Slightly familiar
- ○ Not familiar at all

In your opinion, how feasible is it to pursue a career in global surgery?

- ○ Extremely feasible
- ○ Very feasible
- ○ Moderately feasible
- ○ Slightly feasible
- ○ Not feasible at all

What are in your opinion, obstacles in pursuing a career in global surgery (please select all that apply)?

- ☐ Increased length of training (i.e. F3, fellowships, out-of-programme years)
- ☐ Lack of established career paths in global surgery
- ☐ Lack of surgical role models and mentorship in global health
- ☐ Constrained time to travel abroad during career
- ☐ Difficulty with providing appropriate care for patients living abroad (limited-resources and infrastructure)
- ☐ Ethical issues with providing surgical care in resource-limited communities
- ☐ Financial constraints
- ☐ Other
- ☐ None

Please define what you meant by other?

Thank you for completing the survey.

Survey Powered By Qualtrics
